# Phase Ib study of the oral PI3Kδ inhibitor linperlisib in patients with advanced solid tumors

**DOI:** 10.1007/s10147-024-02657-2

**Published:** 2024-11-13

**Authors:** Jin Li, Junli Xue, Tianshu Liu, Yi Feng, Nong Xu, Jianjin Huang, Yongmei Yin, Jun Zhang, Haibo Mou, Jiangzhong Shentu, Hanying Bao, Zusheng Xu, Zuhong Xu

**Affiliations:** 1 Department of Oncology, East Hospital Affiliated to Tongji University, No. 150 Jimo Road, Pudong New Area, Shanghai, 200120 China; 2 Department of Oncology, Zhongshan Hospital Affiliated to Fudan University, Shanghai, 200032 China; 3 Department of Oncology, The First Affiliated Hospital of Zhejiang University, Hangzhou, 310003 China; 4 Department of Oncology, The Second Affiliated Hospital of Zhejiang University, Hangzhou, 310009 China; 5 Department of Oncology, Jiangsu Provincial People’s Hospital, Nanjing, 210029 China; 6 Department of Oncology, Ruijin Hospital Affiliated to Shanghai Jiao Tong University School of Medicine, Shanghai, 200025 China; 7 Department of Oncology, Zhejiang Shulan Hospital, Hangzhou, 310022 China; 8 Department of Pharmacology, The First Affiliated Hospital of Zhejiang University, Hangzhou, 310003 China; 9 Shanghai Yingli Pharmaceutical Co., Ltd., Shanghai, 201210 China

**Keywords:** Linperlisib, PI3Kδ-selective inhibitor, Advanced solid tumor, Safety

## Abstract

**Background:**

Patients with advanced solid tumors have a suboptimal prognosis. This study investigated the safety and feasibility of linperlisib, a selective phosphatidylinositol 3-kinase delta isoform (PI3Kδ) inhibitor, for treating patients with advanced solid tumors.

**Methods:**

In this phase Ib, single-arm, open-label, multi-center clinical trial, patients with histologically confirmed advanced solid tumors from eight centers in China were enrolled to receive oral linperlisib (80 mg/day). The primary endpoint was safety.

**Results:**

Between August 2019 and June 2022, 94 patients were enrolled in the trial and received the study treatment. The most common (≥ 20%) treatment emergent adverse events (TEAEs) of all grades irrespective of causality were increased aspartate aminotransferase (AST) (26.6%), proteinuria (26.6%), decreased appetite (25.5%), increased alanine aminotransferase (ALT) (22.3%), weight loss (21.3%), and anemia (21.3%). The most common grade ≥ 3 TEAEs were diarrhea (4.3%), increased AST (3.2%), increased ALT (3.2%), neutropenia (3.2%), anemia (3.2%), increased blood alkaline phosphatase (3.2%). The objective response rate (ORR) was 1.1% (95% confidence interval [CI] 0.0–5.8), and the disease control rate (DCR) was 37.2% (95% CI 27.5–47.8). As of the data cutoff, the median follow-up time was 4.2 months (95% CI 2.8–6.9). The median progression-free survival (PFS) was 1.85 months (95% CI 1.79–1.88). The median overall survival (OS) was not reached.

**Conclusion:**

Linperlisib showed an acceptable safety profile and preliminary clinical benefit in patients with a range of advanced solid tumors. Further studies of linperlisib safety and efficacy are warranted.

## Introduction

Phosphatidylinositol 3-kinase (PI3K), a lipokinase composed of regulatory subunit p85 or p101 and catalytic subunit p110, plays a key role in cell proliferation, survival and metabolism by catalyzing phosphatidylinositol 4,5-bisphosphate (PIP2) and phosphorylating phosphatidylinositol 3,4,5-triphosphate (PIP3) which, in turn, activates downstream serine/threonine protein kinase (Akt) [1]. Frequent mutations and amplifications in the *PI3KCA* gene, coupled with the absence of the tumor suppressor gene phosphatase and tensin homolog (*PTEN*) in cancers, underscore PI3K’s significance in tumorigenesis [2, 3]. Among the four PI3K isoforms, PI3Kα and PI3Kβ are widely expressed, whereas PI3Kδ and PI3Kγ are mainly distributed in bone marrow cells. In hematological cancers, PI3Kδ inhibitors are thought to target cancer cells directly, while studies suggest that inhibition of PI3Kδ may additionally promote antitumor immunity in both hematological and solid tumors through preferential T cell suppression. Studies have demonstrated that germline genetic inactivation of PI3Kδ in mice leads to T cell-mediated tumor growth control [4–8]. Following PI3Kδ inhibition, intra-tumoral effector T cells exhibited improved metabolic fitness and enhanced self-renewal capacity. The significant reduction in tumor burden observed in mice was driven by a marked expansion of tumor antigen-specific CD8T cells and resistance to exhaustion as evidenced by decreased programmed cell death protein 1 (PD-1) expression [9]. Inhibition of PI3Kδ leads to decrease in T cells and myeloid-derived suppressor cells, a concomitant increase in effector T cell activity, and reduced growth of multiple tumor types, including 4T1 breast cancer, Lewis lung carcinoma, B16 melanoma, and EL4 thymoma [4].Please confirm the section headings are correctly identified.I have confirmed that it is correct.

Nearly half of patients with hepatocellular carcinoma (HCC) show overexpression of PI3K [10]. Overactivation of the PI3K/Akt pathway enhances the invasive and metastatic capacities of HCC cells. Conversely, inhibition of the PI3K/Akt pathway can induce apoptosis and autophagy in HCC cells [11–13]. Studies have shown that PI-3065, a small-molecule inhibitor of PI3K delta, can suppress survivin expression and directly induce apoptosis of HCC cells, cause mitochondrial toxicity, and inhibit the migration, colony formation, and epithelial to mesenchymal transition abilities of HCC cells, ultimately exerting anti-tumor effects in vivo and in vitro. Furthermore, PI-3065 has also been demonstrated to significantly inhibit growth and metastasis of breast cancer [4, 14].

Linperlisib, a novel oral PI3Kδ small molecule inhibitor, has a different structure from the other existing PI3Kδ inhibitors, providing improved selectivity of PI3Kδ and eliminating PI3Kγ activity. Preclinical data have indicated that linperlisib can induce apoptosis and inhibit the proliferation of malignant B cells and primary tumor cell lines by inhibiting expression of the PI3Kδ protein and reducing the phosphorylation level of the Akt protein. A single-dose 14C-YY-20394 tracer study revealed that linperlisib is primarily excreted through the kidneys [15], whereas duvelisib and idelalisib are predominantly excreted via the intestines. In the phase Ia clinical trial of linperlisib at doses ranging from 20 to 200 mg, linperlisib exhibited relatively good safety and tolerability in patients with relapsed or refractory B-cell hematological malignancies. Although no dose-limiting toxicities were observed at the highest 200 mg/day dose, adverse events (AEs) were noted, and the final recommended phase 2 dose was set at 80 mg/day. Linperlisib demonstrated good antitumor activity with an objective response rate (ORR) of 64% (16/25) (95% confidence interval [CI] 45.2–82.8) and disease control rate (DCR) of 72% (18/25) (95% CI 54.4–89.6) [16]. Based on preclinical findings, linperlisib treatment in the CT26 cancer model reduced tumor volume. As such, we conducted this phase Ib clinical study to explore the safety and feasibility of linperlisib for treatment of advanced solid tumors.

## Patient and methods

### Study design and participants

This phase Ib, single-arm, open-label, multi-center clinical trial recruited patients with advanced or metastatic solid tumors from eight sites in China.

Eligible patients were aged ≥ 18 years, with histologically or pathologically confirmed advanced solid tumor, disease progression after standard treatment, Eastern Cooperative Oncology Group (ECOG) performance status score of 0–2, and at least one measurable lesion according to Response Evaluation Criteria in Solid Tumors, Version 1.1 (RECIST 1.1) criteria, and adequate organ function. The main exclusion criteria included prior use of PI3K targeted agents and clinically symptomatic central nervous system metastasis or meningeal metastasis.

The study was conducted with the approval of independent ethics committees or institutional review boards of all the study centers and in accordance with the principles of the Declaration of Helsinki, International Conference on Harmonization-Good Clinical Practice, and other applicable regulatory requirements. All patients involved in the study provided written informed consent. The study was registered at clinicaltrials.gov with the identifier NCT04049929.

### Procedure

Patients received oral linperlisib 80 mg once per day, taken with warm water either 1 h prior to or 2 h after a meal every day, in a 28-day cycle. Dose adjustments, including holds and reductions, were mandated for grade 3/4 hematologic and grade 3 non-hematologic toxicity. Treatment with linperlisib continued until disease progression, intolerable toxicity, withdrawal of informed consent, death or any other conditions deemed by the investigator to warrant drug discontinuation.

Physical examination, assessment of ECOG performance status, clinical laboratory evaluations, electrocardiogram, and other safety evaluations were conducted to assess safety. AEs were graded according to the National Cancer Institute Common Terminology Criteria for Adverse Events (CTCAE), Version 5.0. All AEs were recorded from the time of signing the informed consent to at least 30 days after the last dose, and were followed until resolution or stabilization. Patients underwent radiographic assessments every 8 weeks by enhanced computed tomography or magnetic resonance imaging according to RECIST 1.1.

### Endpoints

The primary endpoint was safety as assessed by monitoring the frequency, duration, and severity of AEs. Secondary endpoints were efficacy endpoints, including ORR, DCR, and progression-free survival (PFS).

### Statistical analysis

Baseline data are presented descriptively. ORR is determined by the proportion of patients who achieve a confirmed complete response (CR) or partial response (PR) as the best overall response, and is presented with CIs. PFS and overall survival (OS) curves are generated using the Kaplan–Meier method. The incidence of AEs and serious AEs (SAEs) are reported as numbers and percentages.

## Results

### Patients

**Fig. 1:**
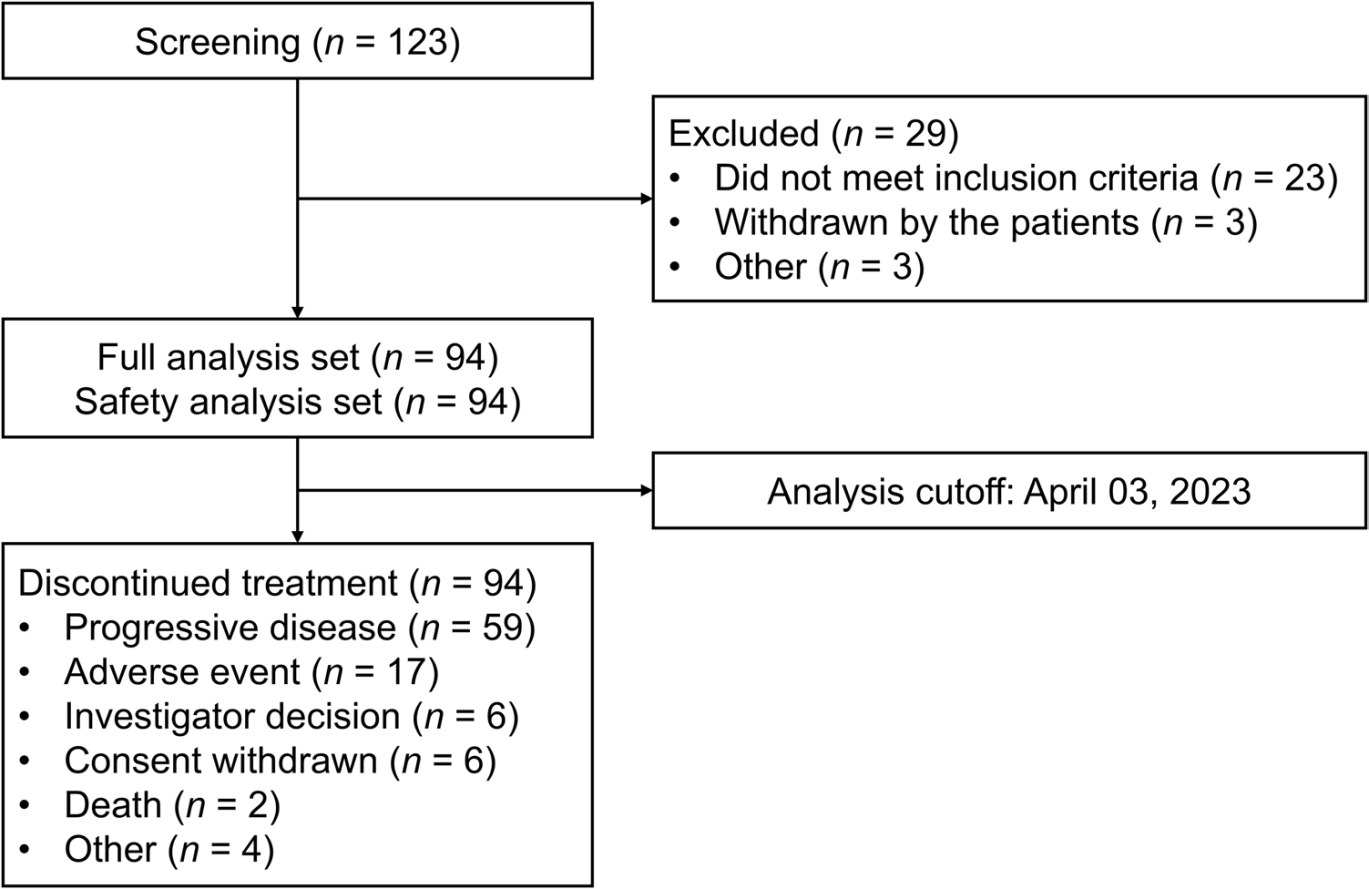
Patient disposition

Between August 2019 and June 2022, a total of 94 eligible patients with advanced solid tumors received at least one dose of linperlisib. The predominant tumor types were intestinal cancer, thymic carcinoma, breast cancer and non-small cell lung cancer (Table 1). At the data cutoff on April 03, 2023, with a median follow-up time of 4.2 months (95% CI 2.8–6.9), all 94 patients had discontinued treatment, primarily due to progressive disease (59, 62.8%) or AEs (20, 21.3%). Two patients (2%) discontinued treatment due to tumor progression leading to death. Seven patients discontinued treatment for other reasons, including use of new anti-tumor therapy (3, 3%), use of drugs prohibited in clinical trials (2, 2%), and self-discontinuation of study drug (1, 1%), and no clinical benefit assessed by the investigator (1, 1%) (Fig. 1).

**Table 1:** Baseline demographics and disease history

Characteristic	*n* (%)
Number of patients	94
Age, median years (min, max)	54.0 (24.0, 75.0)
≥ 65 years, *n* (%)	16 (17.0)
< 65 years, *n* (%)	78 (83.0)
Gender, *n* (%)	
Male	52 (55.3)
Female	42 (44.7)
ECOG PS, *n* (%)	
0	14 (14.9)
1	80 (85.1)
Time since diagnosis, median months (min, max)	30.5 (0.4, 135.0)
Stage, *n* (%)	
III	4 (4.3)
IV	90 (95.7)
Previous therapy, *n* (%)	
Radiotherapy	51 (54.3)
Systemic anticancer therapy	91 (96.8)
Median lines of prior systemic therapy (range)	3 (0–11)
Prior lines of therapy, *n* (%)	
0	3 (3.2)
1	17 (18.1)
2	21 (22.3)
≥ 3	53 (56.4)
Tumor type, *n* (%)	
Colorectal cancer	24 (25.5)
Thymic carcinoma	16 (17.0)
Sarcoma	9 (9.6)
Breast cancer	8 (8.5)
Non–small cell lung cancer	7 (7.4)
Renal cell carcinoma	5 (5.3)
Gastric cancer	4 (4.3)
Nasopharyngeal carcinoma	3 (3.2)
Gallbladder cancer	3 (3.2)
Cholangiocarcinoma	2 (2.1)
Carcinoma of renal pelvis	2 (2.1)
Hepatocellular carcinoma	2 (2.1)
Other^a^	9 (9.6)

^a^Neuroendocrine carcinoma of the lung (*n* = 1); thymoma (*n* = 1); anal carcinoma (*n* = 1); esophageal cancer (*n* = 1); pancreatic cancer (*n* = 1); endometrial cancer (*n* = 1); ovarian cancer (*n* = 1); parotid carcinoma (*n* = 1); oral cavity cancer (*n* = 1)

*ECOG PS* Eastern Cooperative Oncology Group performance status

Sixteen patients (17.0%) were over 65 years old. Most patients (95.7%) had stage IV disease at initial diagnosis. A total of 91 patients (96.8%) had received systemic anti-tumor treatment, with a median of 3 prior treatment lines (Table 1).

### Safety

Ninety-two patients (97.9%) experienced at least one treatment-emergent AE (TEAE), and 43 (45.7%) developed TEAEs of grade 3 or greater. The most common (≥ 20%) TEAEs of all grades irrespective of causality were increased aspartate aminotransferase (AST; 26.6%), proteinuria (26.6%), decreased appetite (25.5%), increased alanine aminotransferase (ALT; 22.3%), weight loss (21.3%), and anemia (21.3%). The most common grade ≥ 3 TEAEs were diarrhea (4.3%), increased AST (3.2%), increased ALT (3.2%), neutropenia (3.2%), anemia (3.2%), increased blood alkaline phosphatase (3.2%). TEAEs, classified by the CTCAE grade and occurring in ≥ 10% of patients, are described in Table 2.

**Table 2:** TEAEs by CTCAE grade (≥ 10%)

TEAE	All grades, *n* (%)	Grade ≥ 3, *n* (%)
*Hematological*		
Anemia	20 (21.3)	3 (3.2)
Leukopenia	16 (17.0)	1 (1.1)
Neutropenia	13 (13.8)	3 (3.2)
*Non-hematological*		
Increased AST	25 (26.6)	3 (3.2)
Proteinuria	25 (26.6)	0
Decreased appetite	24 (25.5)	1 (1.1)
Increased ALT	21 (22.3)	3 (3.2)
Weight loss	20 (21.3)	0
Elevated γ-glutamyltransferase	18 (19.1)	2 (2.1)
Increased blood creatinine	18 (19.1)	2 (2.1)
Increased blood bilirubin	16 (17.0)	1 (1.1)
Nausea	16 (17.0)	0
Increased blood alkaline phosphatase	15 (16.0)	3 (3.2)
Vomiting	15 (16.0)	2 (2.1)
Hypoalbuminemia	15 (16.0)	0
Hyponatremia	15 (16.0)	1 (1.1)
Asthenia	15 (16.0)	0
Rash	14 (14.9)	2 (2.1)
Increased blood lactate dehydrogenase	13 (13.8)	0
Urinary tract infection	13 (13.8)	0
Hypercholesterolemia	12 (12.8)	1 (1.1)
Diarrhea	11 (11.7)	4 (4.3)
Abdominal pain	11 (11.7)	1 (1.1)
Hypertriglyceridemia	10 (10.6)	0
Protein urine present	10 (10.6)	0
Pyrexia	10 (10.6)	0

*ALT* alanine aminotransferase, *AST* aspartate aminotransferase, *CTCAE* Common Terminology Criteria for Adverse Events, *TEAE* treatment-emergent adverse event

Eighty-four patients (89.4%) experienced at least one treatment-related AE (TRAE), with 27 patients (28.7%) having grade 3 or greater events. The most common (≥ 15%) TRAEs were increased AST (22.3%), proteinuria (21.3%), increased ALT (18.1%), decreased appetite (18.1%), and elevated γ-glutamyltransferase (16.0%).

Five patients (5.3%) withdrew from the trial because of TRAEs. Two patients (2.1%) experienced dose reduction of linperlisib due to elevated γ-glutamyltransferase and diarrhea. Eighteen patients (19.1%) suspended linperlisib due to TRAEs and four patients (2.1%) terminated linperlisib due to vomiting, esophagitis, gastritis, or drug eruption. Ten patients (10.6%) reported SAEs related to linperlisib, including diarrhea, vomiting, gastritis, esophagitis, interstitial lung disease, tachypnea, pneumonia, and dermatitis exfoliative generalized. No TRAEs leading to death were reported.

### Pharmacokinetics

**Fig.2:**
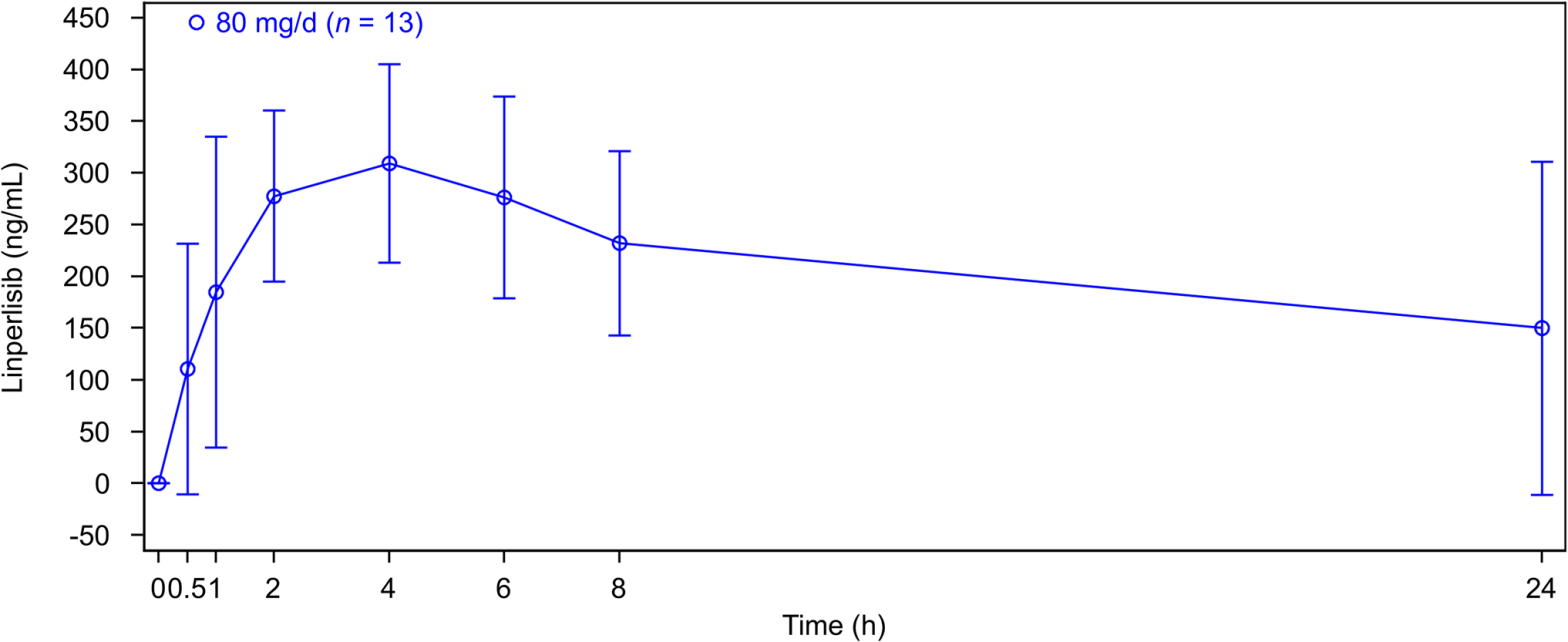
Geometric mean (standard deviation) plasma concentration–time profiles for linperlisib at the 80 mg/day on cycle 1, day 1

Pharmacokinetic analysis included the first 13 patients enrolled in this study who received linperlisib at 80 mg once daily. After a single dose, linperlisib typically reached maximum plasma concentration (C_max_) at 3.9 h (Table 3), followed by a rapid decline in the plasma concentration–time profiles up to 24 h (Fig. 2). The half-life was 14.3 h, and no accumulation was observed after multiple administrations. The trough levels of linperlisib on cycle 1, day 7 were 167.7 ng/ml (range, 68.4–232).

**Table 3:** Linperlisib pharmacokinetic parameters after single dose oral administration

	YY-20394 80 mg qd
	(*n* = 13)
C_max_ (ng/mL), mean (SD)	364.0 (122.0)
T_max_ (h), median (range)	3.9 (1.0, 24.9)
AUC_0-∞_ (h×ng/mL), mean (SD)	7260.0 (4910.0)
AUC_0-24_ (h×ng/mL), mean (SD)	4920.0 (2060.0)
T_1/2_ (h), mean (SD)	14.3 (5.6)
MRT (h), median (range)	18.9 (15.2, 41.3)
CL/F (L/h), mean (SD)	13.5 (4.9)
V_z_/F (L), mean (SD)	251.0 (62.7)

*AUC*_0-24_ area under the curve from time zero to 24 h after start of infusion, *AUC*_0-last_ area under the curve from time zero to extrapolated to infinity, *CL*/*F* Apparent oral clearance, *C*_max_, maximum drug concentration, *qd* once daily, *T*_1/2_, half-life associated with the terminal slope, *T*_max_, time to maximum drug concentration, *V*_z_/*F*, apparent volume of distribution

### Efficacy

**Fig. 3:**
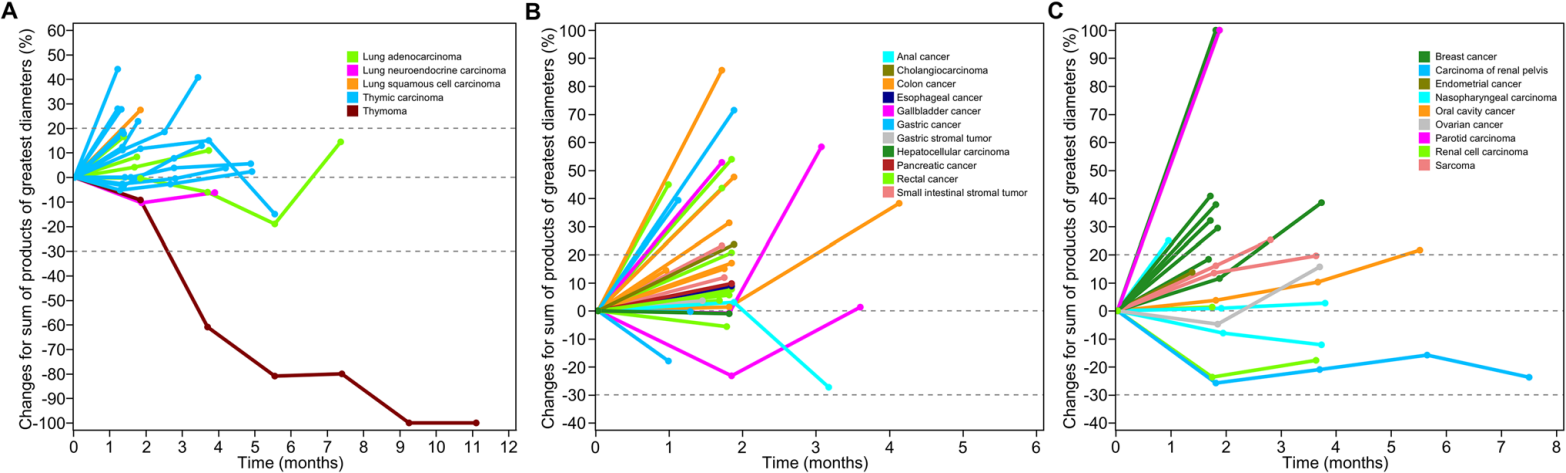
Tumor change from baseline by patient. **A** Lung and mediastinal tumor, **B** gastrointestinal tumor, **C** other types of tumor

**Fig. 4:**
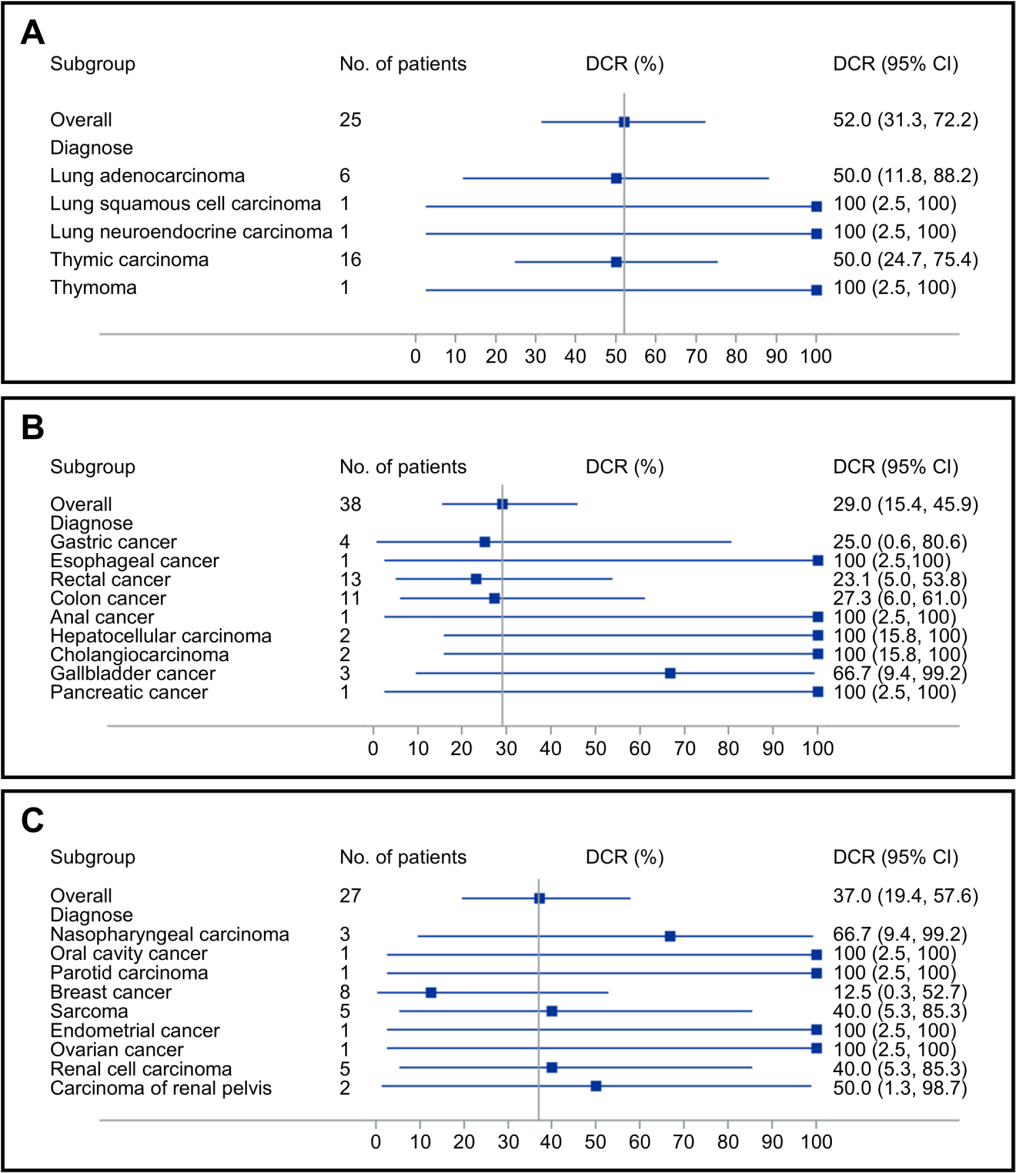
Forest plots of DCR for different tumor types. **A** Lung and mediastinal tumor, **B** gastrointestinal tumor, **C** other types of tumor. *DCR* disease control rate

**Fig. 5:**
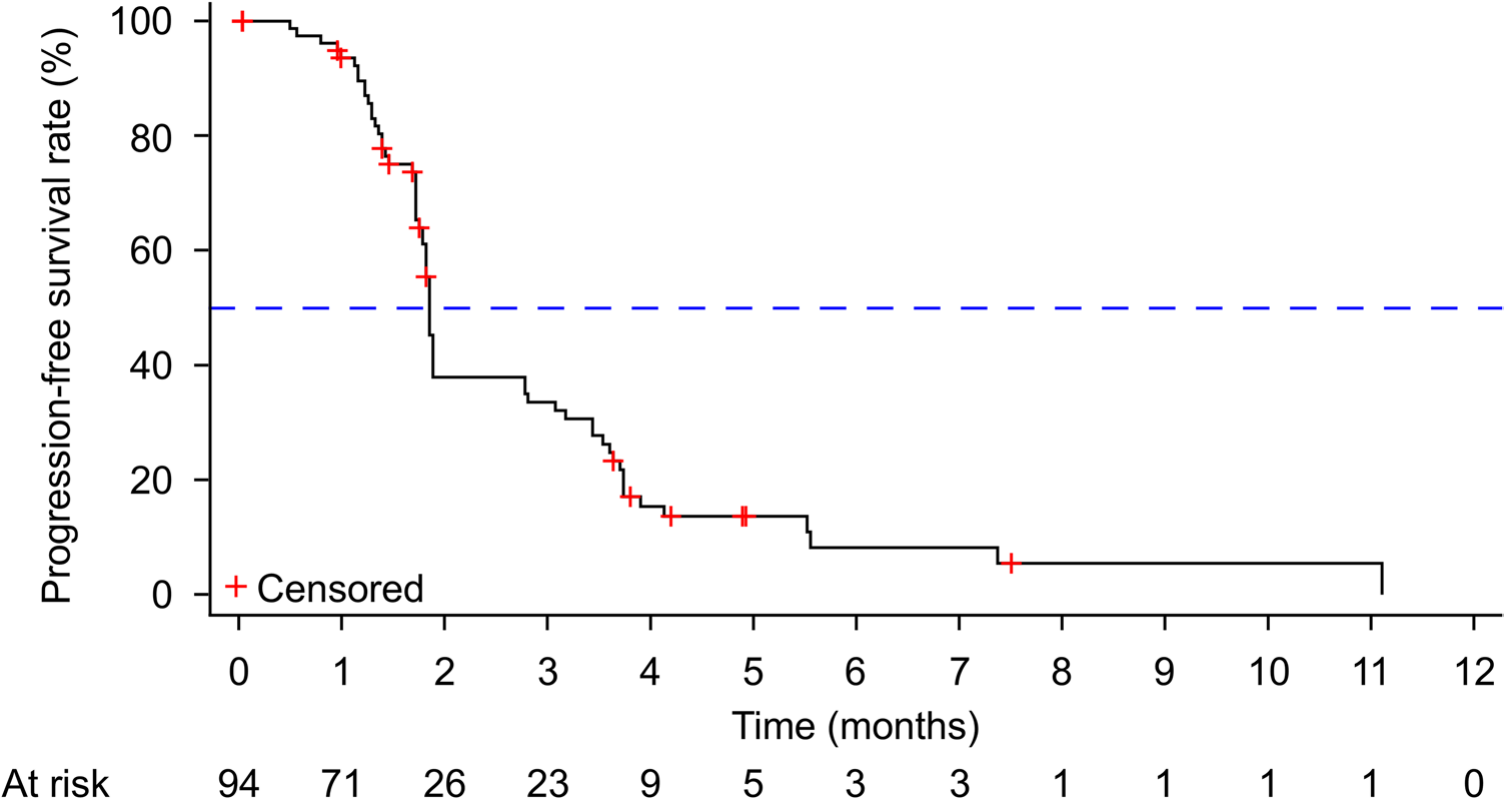
Kaplan–Meier plot of PFS. *PFS* progression-free survival

All 94 patients were included in the efficacy analysis, and the ORR was 1.1% (95% CI 0.0–5.8), with one thymoma patient achieving CR (1.1%). Stable disease was observed in 34 patients, and the DCR was 37.2% (95% CI 27.5–47.8) (Tables 4, 5 and 6; Figs. 3 and 4). Thirty-nine patients had progressive disease as best response and 20 patients had no response assessment after baseline. As of the data cutoff, the median PFS was 1.85 months (95% CI 1.79–1.88) (Fig. 5). The median OS was not reached.

**Table 4:** Lung and mediastinal tumor response

Tumor type	*n*	ORR		DCR	
		*n* (%)	95% CI	*n* (%)	95% CI
Lung adenocarcinoma	6	0 (0.0)	0.0–45.9	3 (50.0)	11.8–88.2
Lung squamous cell carcinoma	1	0 (0.0)	0.0–97.5	0 (0.0)	0.0–97.5
Lung neuroendocrine carcinoma	1	0 (0.0)	0.0–97.5	1 (100.0)	2.5–100.0
Thymic carcinoma	16	0 (0.0)	0.0–20.6	8 (50.0)	24.7–75.3
Thymoma	1	1 (100.0)	2.5–100.0	1 (100.0)	2.5–100.0
Total	25	1 (4.0)	0.1–20.4	13 (52.0)	31.3–72.2

*CI* confidence interval, *DCR* disease control rate, *ORR* objective response rate

**Table 5:** Gastrointestinal tumor response

Tumor type	*n*	ORR		DCR	
		*n* (%)	95% CI	*n* (%)	95% CI
Gastric cancer	4	0 (0.0)	0.0–60.2	1 (25.0)	0.6–80.6
Esophageal cancer	1	0 (0.0)	0.0–97.5	0 (0.0)	0.0–97.5
Rectal cancer	13	0 (0.0)	0.0–24.7	3 (23.1)	5.0–53.8
Colon cancer	11	0 (0.0)	0.0–28.5	3 (27.3)	6.0–61.0
Anal cancer	1	0 (0.0)	0.0–97.5	1 (100.0)	2.5–100.0
Hepatocellular carcinoma	2	0 (0.0)	0.0–84.2	0 (0.0)	0.0–84.2
Cholangiocarcinoma	2	0 (0.0)	0.0–84.2	0 (0.0)	0.0–84.2
Gallbladder cancer	3	0 (0.0)	0.0–70.8	2 (66.7)	9.4–99.2
Pancreatic cancer	1	0 (0.0)	0.0–97.5	1 (100.0)	2.5–100.0
Total	38	0 (0.0)	0.0–9.3	11 (28.9)	15.4–45.9

*CI* confidence interval, *DCR* disease control rate, *ORR* objective response rate

**Table 6:** Other types of tumor response

Tumor type	*n*	ORR		DCR	
		*n* (%)	95% CI	*n* (%)	95% CI
Nasopharyngeal carcinoma	3	0 (0.0)	0.0–70.8	2 (66.7)	9.4–99.2
Oral cavity cancer	1	0 (0.0)	0.0–97.5	1 (100.0)	2.5–100.0
Parotid carcinoma	1	0 (0.0)	0.0–97.5	0 (0.0)	0.0–97.5
Breast cancer	8	0 (0.0)	0.0–36.9	1 (12.5)	0.3–52.7
Sarcoma	9	0 (0.0)	0.0–33.6	3 (33.3)	7.5–70.1
Endometrial cancer	1	0 (0.0)	0.0–97.5	0 (0.0)	0.0–97.5
Ovarian cancer	1	0 (0.0)	0.0–97.5	1 (100.0)	2.5–100.0
Renal cell carcinoma	5	0 (0.0)	0.0–52.2	2 (40.0)	5.3–85.3
Carcinoma of renal pelvis	2	0 (0.0)	0.0–84.2	1 (50.0)	1.3–98.7
Total	31	0 (0.0)	0.0–11.2	11 (35.5)	19.2–54.6

*CI* confidence interval, *DCR* disease control rate, *ORR* objective response rate

## Discussion

**Fig. 6:**
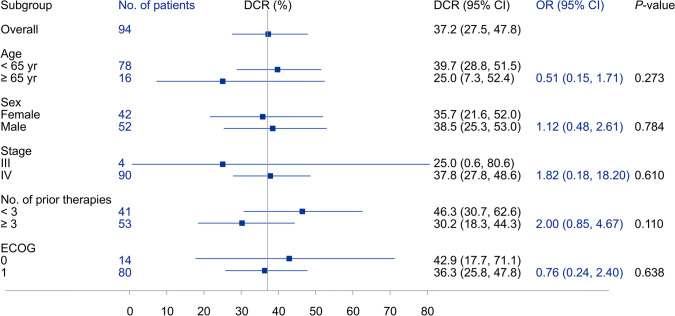
Forest plots of DCR for baseline criteria. *CI* confidence interval, *DCR* disease control rate, *ECOG* Eastern Cooperative Oncology Group, *OR* odds ratio

This phase Ib study evaluated the safety and feasibility of linperlisib in patients with advanced solid tumors. In this study, patients had received a median of three prior systemic treatments, and 56.4% had three or more prior therapies. It is important to note that a high DCR (46.3%) was observed for the linperlisib-treated patients who had less than three prior lines of therapy (Fig. 6). Linperlisib is rapidly absorbed after a single dose and no accumulation is observed after multiple administrations. The pharmacokinetic parameters for 80 mg of linperlisib once daily are similar in advanced solid tumors and B-cell hematological malignancies [16]. Of all patients, 97.9% (92/94) reported TEAEs, with 89.4% (84/94) patients reporting TRAEs. Specifically, 43 (45.7%) patients reported grade 3 or greater TEAEs and 27 (28.7%) patients reported grade 3 or greater TRAEs. Thirty-five SAEs were noted in 29 (30.9%) patients, with 26 (27.7%) patients developing 29 SAEs of grade 3 or greater and 10 (10.6%) patients reporting 12 SAEs related to study drug. The most common (≥ 20%) TEAEs of all grades irrespective of causality were increased AST (26.6%), proteinuria (26.6%), decreased appetite (25.5%), increased ALT (22.3%), weight loss (21.3%), and anemia (21.3%). The most common (≥ 15%) TRAEs were increased AST (22.3%), proteinuria (21.3%), increased ALT (18.1%), decreased appetite (18.1%), and elevated γ-glutamyltransferase (16.0%).

TRAEs occurred in 24 (80.0%) patients received itacitinib plus parsaclisib or parsaclisib monotherapy, with fatigue (30.0%), nausea (23.3%), and anemia (16.7%) the most common. Serious TEAEs related to itacitinib and parsaclisib included one each of fatigue, pain, lung infection, streptococcal bacteremia, malignant neoplasm progression, dyspnea, and pleural effusion [17]. Of the 12 patients received at least one dose of idelalisib 150 mg twice daily, frequent (≥ 25%) TEAEs included pyrexia (50%), increased AST (41.7%), increased ALT (33.3%), and maculopapular rash (25%). Common (≥ 25%) grade ≥ 3 TEAEs were increased AST (41.7%), increased ALT (25%), and maculopapular rash (25%) [18]. The most common TEAEs (all grade), included hyperglycemia (52.4%), fatigue (46.0%), and hypertension (41.3%). The most common grade 3 or 4 TEAEs (≥ 10% combined) were hypertension (30.2%/0), hyperglycemia (22.2%/1.6%), hypophosphatemia (11.1%/0), and lymphocyte count decreased (7.9%/4.8%) [19]. AEs possibly related to copanlisib occurred in 49 patients (86%). The most common (≥ 20%) TRAEs (all grades) included hyperglycemia (63%), nausea (37%), and hypertension (21%). The most common drug-related grade 3 AEs were hyperglycemia (30%), hypertension (14%), and rash (7%) [20].

The TEAEs reported for 80 mg/day linperlisib in this study are generally consistent with the expected TEAE profile of other PI3Kδ inhibitors such as idelalisib, copanlisib, and parsaclisib, with no new or unexpected AEs emerging. The incidence of hyperglycemia related to linperlisib was 2.1% (≥ grade 3, 0%). Linperlisib is a novel PI3Kδ inhibitor that differs from other existing PI3K inhibitors in structure, improving PI3Kδ selectivity and removing PI3Kγ activity. P110α is the primary insulin-responsive PI3K. Compounds targeting p110α block the acute effects of insulin treatment in vivo. Thus, the incidence of hyperglycemia with linperlisib is lower than with other PI3K inhibitors, particularly pan-PI3K inhibitors [21]. It is noteworthy that, unlike other PI3K inhibitors, the incidence of diarrhea, colitis, and hepatotoxicity was very low [22].

Deregulation of the PI3K pathway plays a critical role in the development and progression of cancer, and has been frequently implicated in a wide spectrum of malignancies, including glioma, prostate, breast, ovarian, and endometrial cancer. Alterations of PI3K, due to mutations in its catalytic or regulatory subunits, is observed in a subgroup of TETs, in particular thymic carcinomas. A new cell line (MP57) possesses all the tested markers of thymic epithelial cells, validating it as a genuine thymic carcinoma cell line. Next-generation sequencing analysis of MP57 identified a mutation in the gene *PIK3R2*, which encodes a regulatory subunit of PI3K. Further analysis found different mutations across multiple PI3K subunit genes in another cell line and several primary thymic carcinoma samples, including two catalytic subunits (*PIK3CA* and *PIK3CG*) and another regulatory subunit (*PIK3R4*). Inhibiting PI3K with GDC-0941 resulted in in vitro antitumor activity in thymic epithelial tumors cells carrying mutant PI3K subunits. Targeting PI3K may be an effective strategy to treat these tumors [23]. In this clinical study, the DCR in patients with thymic carcinoma reached 50% and one patient with thymoma (B3) achieved CR as best response. Blocking PI3Kδ activity with a PI3Kδ inhibitor suppressed HCC-cell proliferation and dampened key features of malignant HCC, including the up-regulation of telomerase reverse transcriptase (*TERT*). Mechanistically, H_2_O_2_ induced oxidative modification of the serpin peptidase inhibitor clade A member 3 (*SERPINA3*), blocking its ubiquitin-dependent degradation and enhancing its activity as a transcriptional activator of PI3Kδ and *TERT*. PI3Kδ inhibition is a potential treatment for HCC [24]. A total of two patients with HCC were enrolled in this study, and one of them had shrinkage of target lesions.

In the first-in-human study of copanlisib, of 48 patients with advanced solid tumors, one patient with endometrial carcinoma exhibiting both *PIK3CA* and *PTEN* mutations and complete *PTEN* loss achieved a CR, two metastatic breast cancer patients had a PR, and 15 patients achieved stable disease [20]. In the Phase 1 study of copanlisib, among 10 Japanese patients with advanced or refractory solid tumors, no patients achieved a CR or PR, and DCR was 40.0%. The median PFS was 52 days (95% CI 21–82), and the 3-month PFS rate was 12% (95% CI 1–40) [25]. In the Phase 1 studies of parsaclisib monotherapy, of five patients with advanced solid tumors, none achieved a CR or PR, and the DCR was 2% [17].

In this study, 95.7% of patients with stage IV solid tumors were enrolled, of whom 54.3% were pre-treated with radiotherapy, 96.8% with systemic anti-tumor therapy, and 56.4% received 3 or more lines of prior systemic anti-tumor therapy. The ORR was 1.1% (95% CI 0.0–5.8), with 1 patient with thmoma (B3) achieving CR (1.1%). The DCR was 37.2% (95% CI 27.5–47.8). As of the data cutoff, the median PFS was 1.85 months (95% CI 1.79–1.88). In thymoma, gallbladder cancer, anal canal squamous cell carcinoma, renal cell carcinoma, and renal pelvis cancer, target lesions had shrunk by more than 20%. However, due to the small sample size for each tumor type, further clinical trials are required for validation. Currently, PI3Kδ inhibitors have shown good tolerance and efficacy in the treatment of B-cell hematological malignancies. However, their application in treating solid tumors remains limited, and is mostly in combination with chemotherapy or programmed death-ligand 1 (PD-L1) inhibition [17, 26, 27].

This manuscript has several limitations that should be taken into consideration. Firstly, the study design was limited to a single-arm, which may affect the generalizability of the results. Secondly, the follow-up period was short, and the median OS was not reached, which may limit the ability to draw conclusions about the long-term efficacy of the treatment. Further studies with larger sample sizes and longer follow-up periods are needed to confirm these findings. Finally, although the safety results for 80 mg/day of linperlisib was better in advanced solid tumors than in B-cell hematological malignancies [16], we still set the dose at 80 mg/day considering that we mainly use combination therapy in advanced solid tumors. On the other hand, we could explore dose higher than 80 mg/day to seek better efficacy in subsequent studies of advanced solid tumors with linperlisib combined with standard of care. Multiple investigator-initiated phase 2 clinical studies of linperlisib combined with standard of care are conducted to explore the tumor type and dose in advanced renal cancer, urothelial cancer, biliary tract tumor, metastatic castration-resistant prostate cancer and non-small cell lung cancer.

## Conclusion

Linperlisib showed an acceptable safety profile and preliminary clinical benefit in patients with a range of advanced solid tumors. Further studies of linperlisib safety and efficacy are warranted.

## Data Availability

The datasets used and analyzed during the current study are available from the corresponding author on reasonable request.
